# Long chain ceramides raise the main phase transition of monounsaturated phospholipids to physiological temperature

**DOI:** 10.1038/s41598-022-25330-y

**Published:** 2022-12-02

**Authors:** Hiroshi Takahashi, Tomohiro Hayakawa, Asami Makino, Kunihiko Iwamoto, Kazuki Ito, Satoshi B. Sato, Toshihide Kobayashi

**Affiliations:** 1 Lipid Biology Laboratory, RIKEN, Wako, Saitama 351-0198 Japan; 2 Biophysics Laboratory, Division of Pure and Applied Science, Graduate School of Science and Technology, Gunma University, Maebashi, 371-8510 Japan; 3 RIKEN SPring-8 Center, Sayo, Hyogo 679-5148 Japan; 4 Department of Biophysics, Graduate School of Science, Kyoto University, Kyoto, 606-8502 Japan; 5 UMR 7021 CNRS, Université de Strasbourg, 67401 Illkirch, France

**Keywords:** Biochemistry, Biophysics

## Abstract

Little is known about the molecular mechanisms of ceramide-mediated cellular signaling. We examined the effects of palmitoyl ceramide (C16-ceramide) and stearoyl ceramide (C18-ceramide) on the phase behavior of 1-palmitoyl-2-oleoyl-*sn*-glycero-3-phosphocholine (POPC) and 1-palmitoyl-2-oleoyl-*sn*-glycero-3-phosphoethanolamine (POPE) using differential scanning calorimetry (DSC) and small- and wide-angle X-ray scattering (SAXS, WAXS). As previously published, the presence of ceramides increased the lamellar gel-to-lamellar liquid crystalline (L_β_–L_α_) phase transition temperature of POPC and POPE and decreased the L_α_-to-inverted hexagonal (L_α_–H_II_) phase transition temperature of POPE. Interestingly, despite an ~ 30° difference in the main phase transition temperatures of POPC and POPE, the L_β_–L_α_ phase transition temperatures were very close between POPC/C18-ceramide and POPE/C18-ceramide and were near physiological temperature. A comparison of the results of C16-ceramide in published and our own results with those of C18-ceramide indicates that increase of the carbon chain length of ceramide from 16 to 18 and/or the small difference of ceramide content in the membrane dramatically change the phase transition temperature of POPC and POPE to near physiological temperature. Our results support the idea that ceramide signaling is mediated by the alteration of lipid phase-dependent partitioning of signaling proteins.

## Introduction

Ceramide is a central intermediate of sphingolipid metabolism and is a signaling lipid involved in a number of pathophysiological phenomena^1,2^. Ceramide is synthesized de novo in the cytoplasmic leaflet of the ER and metabolized to glucosylceramide (GlcCer), a precursor of more-complicated glycosphingolipids^3^. Alternatively, ceramide is transported to the *trans* Golgi network (TGN) by ceramide transfer protein (CERT) or by vesicular traffic for sphingomyelin (SM) biosynthesis in the lumen of TGN^4^. SM and glycosphingolipids are then transported to the plasma membrane. From the plasma membrane, SM and glycosphingolipids are endocytosed to late endosomes/lysosomes and degraded to ceramide. Acid sphingomyelinase (aSMase) is involved in the degradation of SM in late endosomes/lysosomes and on the outer leaflet of the plasma membrane after its secretion, whereas neutral sphingomyelinases (nSMases) digest SM in the cytoplasmic leaflets of plasma membrane and intracellular organelles^2^. Recent results also indicate the CERT-mediated transport of ceramide to multivesicular bodies^5,6^. Thus, ceramide can be formed both in the outer/luminal and inner/cytoplasmic leaflet of cellular membranes. In addition, spontaneous rapid flip-flop of ceramide^7–9^ equilibrates ceramide between inner and outer leaflets.

Ceramide synthesis in mammals is catalyzed by a family of six ceramide synthases (CerS1-6). These enzymes transfer fatty acyl coenzyme A of different chain lengths to the amino group of a sphingosine base^10,11^. CerS1 exclusively uses long chain 18 carbon fatty acid, forming C18-ceramide. On the other hand, CerS5 and CerS2 are involved in the formation of long chain C16-ceramide and very long chain C20-26-ceramides, respectively^10,11^. Evidence of the different physiological functions of different ceramide molecular species is accumulating. Even two carbon chain differences in long chain ceramide produce different effects. For example, C16-ceramide antagonizes the insulin receptor and induces insulin resistance^11,12^. In contrast, C18-ceramide is not involved in insulin resistance but regulates fatty acid oxidation^13^. It was also observed that learning in the Morris water maze test is associated with an increase in the concentration of C18-ceramide, but a decrease in the levels of C16-SM and C24:1-SM in the hippocampus of trained mice compared to yoked controls^14^. Among various ceramides, only C18-ceramide was selectively downregulated in head and neck cancer tissues^15^.

Little is known about the molecular mechanisms of ceramide-mediated cellular signaling^2,16–18^. The membrane concentration of ceramide is low in the steady state (0.1–1% of total phospholipids)^19^. However, activation of SMases induces ceramide-enriched membrane domains^20–25^. Ceramides are extremely hydrophobic and increase the molecular order of phospholipids in membranes. Ceramides also induce phase separation, domain formation, pore formation, and flip-flop of phospholipids in membrane bilayers^17,26,27^. It is postulated that these physical properties are involved in the cellular functions of ceramides. However, the relationship between the physiological role of ceramides and the physical properties of ceramide-containing membranes is not well understood^17,26,27^.

Lipids are asymmetrically distributed in the cellular membrane bilayer. Phosphatidylcholine (PC) and SM are mainly distributed in the outer leaflet of the plasma membrane, whereas the inner membrane is enriched with phosphatidylethanolamine (PE) and phosphatidylserine (PS)^28–30^. A small amount of SM is also distributed in the inner leaflet, where the lipid forms specific domains^29^. The effect of long chain ceramides (C16-ceramide and C18-ceramide) on the membrane organization of outer leaflet mimic (PC) and inner leaflet mimic (PE) artificial membrane systems has been examined. Since long chain ceramides exhibits very high main phase transition temperatures (C16-ceramide (90–93.2 °C), C18-ceramide (92.2 °C))^31,32^, the presence of ceramides is expected to induce significant alterations in the biophysical properties of phospholipid membranes.

Both monolayer^33^ and bilayer^27,34–39^ model systems have been employed to study the phase behavior of PC/long chain ceramide membranes. By multiprobe approach, Silva et al. reported the complete phase diagram of the 1-palmitoyl-2-oleoyl-*sn*-glycero-3-phosphocholine (POPC)/C16-ceramide system^40^. The group also examined the effect of different acyl chain ceramides on POPC^41^. The published results indicate that saturated ceramides have a strong impact on the fluid PC membrane, increasing its order and promoting gel/fluid phase separation and that the differences between saturated ceramides are mainly related to the morphology and size of the gel domains. Very long chain ceramide (C24-ceramide) induced deformation of the membrane^41^.

Compared to the PC/ceramide system, a limited number of articles have been published on the phase behavior of PE/ceramide. Previously, Alonso and Goni’s group showed that the addition of egg ceramide (with C16-ceramide as the main molecular species) and C16-ceramide increased the lamellar gel-to-lamellar liquid crystalline (L_β_–L_α_) phase transition temperature and decreased the lamellar liquid crystalline-to-inverse hexagonal (L_α_-H_II_) phase transition temperature of non-natural 1,2-dielaidoyl-*sn*-glycero-3-phosphoethanolamine (18:1(Δ9-trans)PE)^42,43^ using differential scanning calorimetry (DSC), ^31^P nuclear magnetic resonance (NMR) and small-angle X-ray scattering (SAXS). Recently, Doroudgar and Lafleur examined the effect of C16-ceramide on the lipid polymorphism of 1-palmitoyl-2-oleoyl-*sn*-glycero-3-phosphoethanolamine (POPE) using DSC and sequential ^2^H and ^31^P NMR^44^. Their results also indicated that the presence of C16-ceramide led to an upshift of the L_β_–L_α_ phase transition temperature and to a downshift of the L_α_-H_II_ transition temperature. The effect of C18-ceramide on PE has not been examined.

Here, we examined the effects of C18-ceramide on POPC and POPE. POPC is a major PC in mammalian cells and is the most abundant molecular species of PC in the plasma membrane of human fibroblasts^45^ and mouse liver^46^. POPE is also enriched in the plasma membrane of human skin fibroblasts^45^.

As expected, the presence of ceramide increased the L_β_–L_α_ phase transition temperature of POPC and POPE, and decreased the L_α_-H_II_ transition temperature of POPE. Interestingly, despite the large difference in the main phase transition temperatures of POPC (− 7 to − 2 °C^47–50^) and POPE (25 °C^44,51–54^), the presence of a small amount (5%) of C18-ceramide raised the main phase transition of both lipids to physiological temperature (35–40 °C). Comparison of the results of C16-ceramide in published and our own results indicates that the increase of the chain length of ceramide from 16 to 18 or the small difference of ceramide content in the membrane dramatically change the phase transition temperature of the lipid membrane to near physiological temperature.

## Results

### Differential scanning calorimetry (DSC) measurements

**Figure 1:**
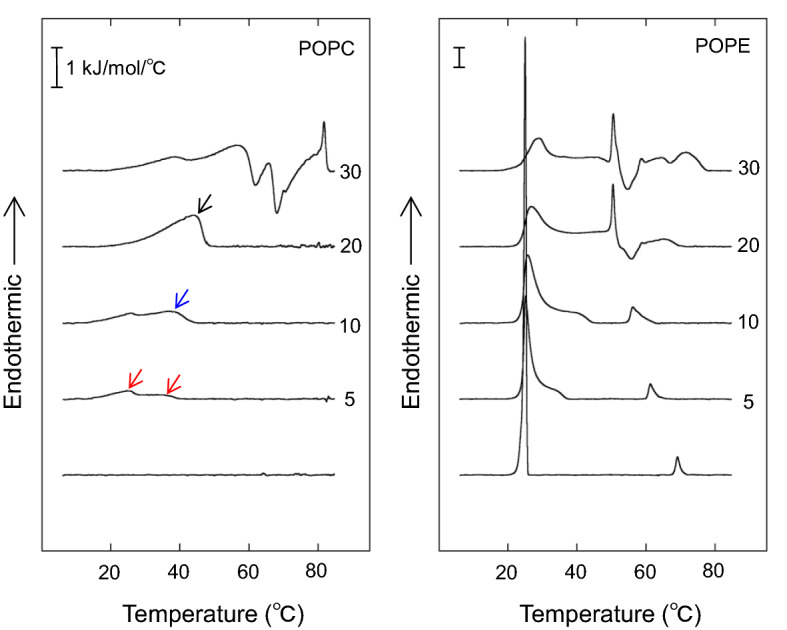
DSC heating thermograms of 1 mM POPC and POPE containing different amounts of C18-ceramide (0–30 mol%). The thermograms were obtained from samples dispersed in 20 mM HEPES buffer (pH 7.0) containing 100 mM NaCl and 10 mM EDTA, and were recorded at scan rate of 0.5°/min.

We examined the effect of C18-ceramide on the DSC thermograms of POPC and POPE over the temperature range of 5–85 °C (Fig. 1). POPC exhibits a L_β_–L_α_ main phase transition at approximately − 7 to − 2 °C^47–50^ and thus does not show a phase transition under the temperature conditions of Fig. 1. In contrast, when POPC containing 5 mol% of C18-ceramide was examined, weakly energetic broad endothermic peaks were observed at ~ 25 °C and ~ 35 °C (red arrows). A broad peak at higher temperature centered at ~ 40 °C appeared (blue arrow) in the presence of 10 mol% C18-ceramide. This higher temperature transition became more energetic and centered at ~ 45 °C (black arrow) in the presence of 20 mol% C18-ceramide. The profiles of these thermograms are similar to the previous results of the POPC/brain ceramide system^47^ and POPC/C16-ceramide^40^, except that C18-ceramide exhibited a phase transition at higher temperatures. It is proposed that the observed endotherm indicates the existence of ceramide-rich domains^40,47,55^.

POPE exhibited transitions of L_β_–L_α_ (ΔH: 21.9 kJ/mol) and L_α_–H_II_ (ΔH: 1.67 kJ/mol) at 25.0 and 72 °C, respectively (Fig. 1). These values are consistent with published results^44,51–54,56^. The onset temperature of the L_β_–L_α_ transition was unaltered in the presence of 5 mol% C18-ceramide. However, the transition peak broadened and an additional broad peak appeared at ~ 33 °C (the total ΔH corresponding to these peaks was 18.9 kJ/mol). This peak shifted to ~ 40 °C in the presence of 10 mol% C18-ceramide (ΔH: 25.1 kJ/mol). The transition temperature of L_α_–H_II_ of POPE was significantly decreased with increasing C18-ceramide. The peak of the L_α_–H_II_ transition became broad and asymmetric. The transition peak of L_α_–H_II_ merged with the L_β_–L_α_ transition in the presence of 20 mol% C18-ceramide. In addition, an exotherm and endotherm were observed at ~ 50–58 °C and 58–70 °C, respectively, for POPE/20 mol% C18-ceramide, suggesting the appearance of the solid state of C18-ceramide, as observed more evidently for the POPC/30 mol% C18-ceramide system. For POPE/30 mol% C18-ceramide, the endotherm and exotherm became more energetic above ~ 50 °C, while the temperature ranges of the endotherms of L_β_–L_α_ and L_α_–H_II_ were essentially unaltered.

The onset temperature of the L_β_–L_α_ transition of POPE/C18-ceramide systems was almost constant in the POPE system containing 0, 5, and 10 mol% of C18-ceramide (Table 1). This result indicates that the L_β_ and L_α_ phases coexist below the L_β_–L_α_ transition temperature according to the Gibbs phase rule^57^. In contrast, the onset transition peak of L_α_–H_II_ was significantly reduced by increasing the C18-ceramide contents from 0 to 10 mol %, indicating that POPE and C18-ceramide mix in the fluid bilayer phase^57^.

**Table 1:** The L_β_–L_α_ and L_α_-H_II_ phase transition temperatures of POPC/C18-ceramide dispersions and POPE/C18-ceramide dispersions.

	L_β_–L_α_ phase transition	L_α_–H_II_ phase transition
	Onset/end (°C)	Onset/end (°C)
**POPC/C18-ceramide (molar ratio)**		
100:0	–	–
95:5	12.7/41.4	–
90:10	12.7/45.0	–
**POPE/C18-ceramide (molar ratio)**		
100:0	21.1/26.0	67.3/72.0
95:5	21.1/37.6	59.4/66.3
90:10	21.1/45.2	53.8/63.0

### Small-angle X-ray scattering (SAXS) measurements

**Figure 2:**
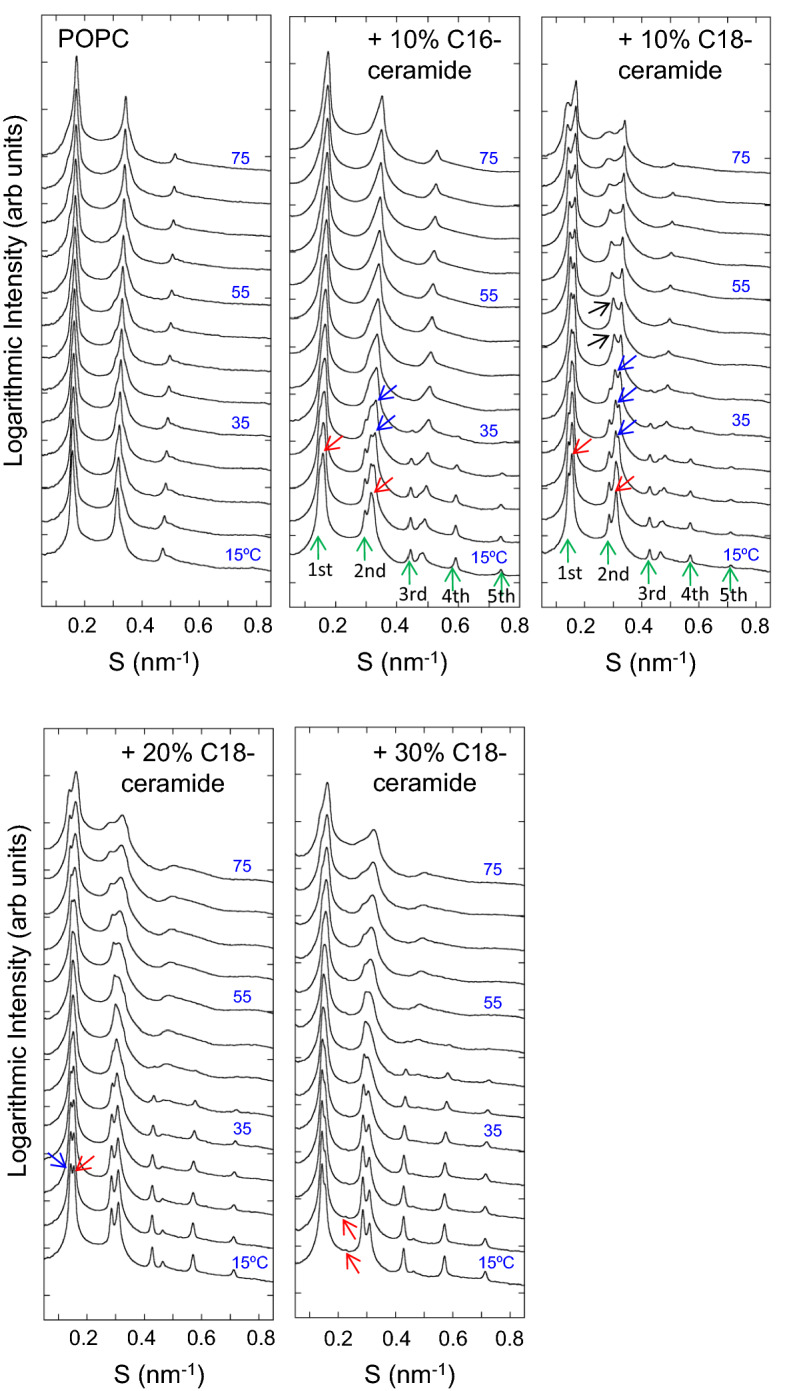
Small-angle X-ray scattering (SAXS) profiles obtained from POPC, POPC/10% C16-ceramide and POPC/C18-ceramide containing different amounts of C18-ceramide (10, 20, 30 mol%). Data were taken from 15 to 75 °C at 5° interval. All samples (150 mM final lipid concentration) were dispersed in 20 mM HEPES buffer (pH 7.0) containing 100 mM NaCl and 10 mM EDTA.

SAXS provides information on the structure of lipids at each temperature. SAXS of POPC exhibits lamellar patterns throughout the temperature range of 15–75 °C (Fig. 2). The lamellar repeat distance (*d*-spacing) of POPC was 6.36 nm at 15 °C. This value decreased as the temperature increased and reached 5.84 nm at 75 °C. Pabst et al.^58^ reported that the *d*-spacings of POPC dispersed in pure water become larger with increasing temperature above 40 °C. They interpreted this behavior as an increase in thermal fluctuations due to the rise in temperature, resulting in stronger steric repulsion between the adjacent bilayers. In other words, increasing the temperature reduces the rigidity modulus of POPC bilayers in pure water. A recent study revealed that the interactions of monovalent ions Na^+^ and Cl^−^ with the neutral head groups of a phospholipid increase the bending rigidity of the phospholipid bilayers^59^. In our study, POPC was dispersed in 20 mM HEPES buffer containing 100 mM NaCl and 10 mM EDTA. Under the experimental conditions, the bending modulus of the POPC bilayer is suppressed to a lower level, and the increase in the thickness of the water layer due to increased repulsive interaction between the bilayers is not significant. Instead, the decrease in bilayer thickness is more predominant, resulting in decreased *d*-spacing with increasing temperature. The first- and second-order lamellar reflections of POPC showed shoulders at the smaller or wider angle side, depending on the temperature. Since the buffer solution contained 100 mM NaCl in our experiments, the observed broadening of the peaks may be attributed to the cation-induced phase separation reported for the POPC L_α_ phase^60,61^.

The SAXS of POPC/10 mol% C16-ceramide and POPC/10 mol% C18-ceramide are compared in Fig. 2. In both mixtures, two sets of lamellar reflections were observed at 15 °C, clearly indicating the existence of the two phases. The shorter *d*-spacings of the peaks indicated by red arrows were 6.26 nm and 6.44 nm for POPC/10 mol% C16-ceramide and POPC/10 mol% C18-ceramide, respectively. Up to a 5th-order reflection was observed for a lamellar structure with a longer *d*-spacing (green arrows). The reflection intensity from this longer lamellar structure decreased with increasing temperature and disappeared at 40 °C in POPC/10 mol% C16-ceramide and at 45 °C in POPC/10 mol % C18-ceramide. At lower temperatures, the positions of the diffraction peaks were almost constant. This lamellar structure with a larger *d*-spacing is considered to indicate the ceramide-rich phase separated region. C18-ceramide showed a slightly larger *d*-spacing, indicating that the C18-ceramide-rich phase exhibits slightly thicker membrane domains than the C16-ceramide-rich domains. In contrast, the lamellar structure with the smaller *d*-spacing at 15 °C is attributed to the ceramide-poor region. The *d*-spacing of this lamellar structure is larger than that of pure POPC (6.36 nm), indicating the presence of ceramide in this membrane region. Due to the long saturated hydrocarbon chain of ceramide, which has a high phase transition temperature^32^, the membrane thickness of the ceramide-rich domain is postulated to be larger than that of the POPC-rich region. For the reflection peaks from the ceramide-poor region (smaller *d*-spacing), peak splitting into two was observed above 20 °C (blue arrows). In the case of POPC/10 mol % C18-ceramide, as clearly observed in the second-order peak, the split into two peaks shifted to opposite angle sides by increasing temperature, and the peak at the smaller angle side appeared to merge with the peak derived from the ceramide domain at 40–45 °C (black arrows). Such peak splitting (phase separation) was clearly observed up to 75 °C. In the case of POPC/10 mol % C16-ceramide, two peaks became a single broad peak above 45 °C. Since the phase transition temperature (Tm) values of C16-ceramide and C18-ceramide are almost identical^32^, the different effects of C16-ceramide and C18-ceramide may be due to the hydrophobic mismatch between C18-ceramide and POPC.

Similar to POPC/10 mol% C18-ceramide, two sets of lamellar peaks that originated from the C18-ceramide-rich and -poor regions were observed at 15 °C for 20 mol% C18-ceramide (Fig. 2). The *d*-spacings of these lamellar structures at 15 °C were 6.44 (red arrow) and 7.01 (blue arrow) nm. These values were slightly larger than those of POPC/10 mol% C18-ceramide, presumably due to the increased C18-ceramide concentration in the membranes. Compared to POPC/10 mol% C18-ceramide, the intensity of the reflections from the C18-ceramide-rich region was greater than that of the C18-ceramide-poor region, as expected, and the reflection peak positions were almost constant up to ~ 45 °C. The reflection peaks from the C18-ceramide-poor region were shifted to the smaller angle region by the temperature increase and appeared to merge with the peaks of the C18-ceramide-rich region at 45 °C. Although a clear splitting of the peaks derived from the C18-ceramide-poor region was not detected, the peaks from the C18-ceramide-poor region became broad, with a shoulder peak on the wider angle side, upon increasing the temperature. Above 50 °C, this shoulder peak became clear. Although the SAXS peaks of POPC/20 mol% C18-ceramide at high temperatures (~ 60–75 °C) were broader than those of POPC/10 mol% C18-ceramide, their overall profiles were similar.

At 30 mol% C18-ceramide (Fig. 2), the SAXS patterns were similar to those of POPC/20 mol% C18-ceramide. The *d*-spacings of the C18-ceramide-rich and C18-ceramide-poor regions at 15 °C were 6.46 nm and 7.02 nm, respectively, and were slightly larger than those of POPC/20 mol% C18-ceramide. The intensity of reflections from the C18-ceramide-rich region became greater than those of the C18-ceramide-poor region, suggesting an increase of the C18-ceramide-rich region. A small reflection at 4.42 nm (S = 0.226 nm^−1^) (red arrow) was observed at 30 mol% C18-ceramide. According to previous reports^43,55^, this reflection is suggested to originate from C18-ceramide clusters or aggregate phases separated from the POPC membrane.

**Figure 3:**
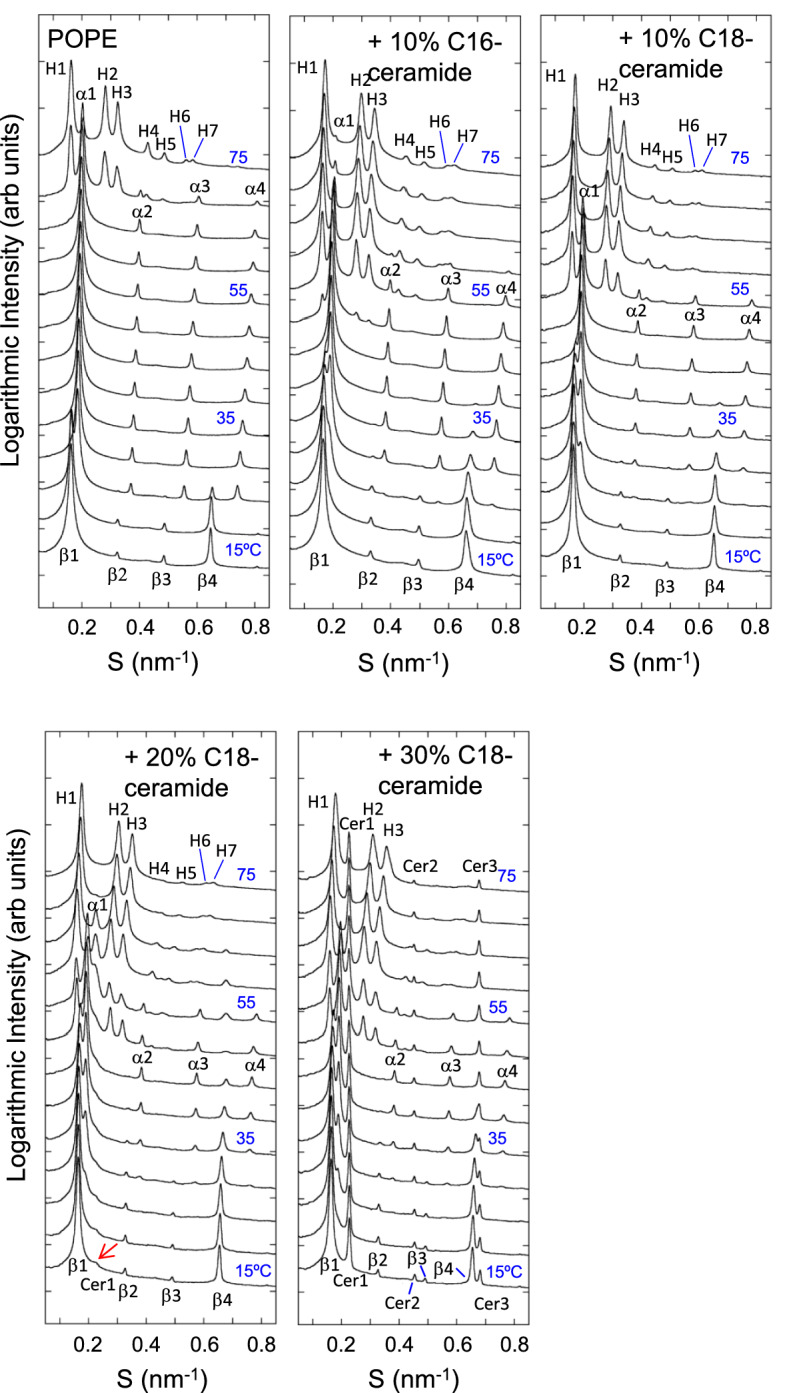
Small-angle X-ray scattering (SAXS) profiles obtained from POPE, POPE/10% C16-ceramide and POPE/C18-ceramide containing different amounts of C18-ceramide (10, 20, 30 mol%). Data were taken from 15 to 75 °C at 5° interval. All samples (150 mM final lipid concentration) were dispersed in 20 mM HEPES buffer (pH 7.0) containing 100 mM NaCl and 10 mM EDTA.

The SAXS profiles of pure POPE are shown in Fig. 3. Following Rappolt et al.^52^, the first to fourth-order lamellar reflection peaks of the L_α_ and L_β_ phases of POPE are denoted as α1–α4 and β1–β4, respectively (Fig. 3). Similarly, the peaks originating from the H_II_ phase are denoted in order from the small-angle side as H1, H2, etc. The *hk* miller indices of the H_II_ phase peaks were attributed according to Rappolt et al.^52^ as follows: H1 (1,0), H2 (1,1), H3 (2,0), H4 (2,1), H5 (3,0), H6 (2,2) and H7 (3,1). POPE was in L_β_ at 15 and 20 °C. At 25 °C, α1–α4 peaks appeared. At 30 °C, only L_α_ signals were observed. This continued until 65 °C. At 70 and 75 °C, L_α_ and H_II_ coexisted. In the presence of 10% C16-ceramide, β2 and β3 signals were observed from 15 to 30 °C and the β4 peak was observed up to 40 °C. The β1 peak displayed a shoulder next to the α1 peak at 40 °C. H_II_ phases appeared at 50 °C, and L_α_ and H_II_ coexisted up to 75 °C. Similar to C16-ceramide, in the presence of 10% C18-ceramide, β2 and β3 signals were observed from 15 to 30 °C and the β4 peak was observed up to 40 °C. The β1 peak was still split from the α1 peak at 40 °C. Unlike C16-ceramide, H_II_ phase was not detected at 50 °C. L_α_ and H_II_ coexisted at 55 and 60 °C. Above 65 °C, only the H_II_ was observed. For 10% C16-ceramide and C18-ceramide at 15 °C, the lamellar repeat *d*-spacings were 6.06 and 6.14 nm, respectively. These values were slightly smaller than that of pure POPE (6.19 nm). At 45 °C, 10% C16-ceramide and C18-ceramide exhibited *d*-spacings of 5.13 nm and 5.22 nm, respectively, while the value of pure POPE was 5.17 nm. Alteration of *d*-spacing indicates the coexistence of POPE and ceramide. No clear peak splitting or broadening of the peaks was observed for the POPE/10 mol% C16-ceramide or C18-ceramide system.

For POPE/20 mol% C18-ceramide, the *d*-spacing of the L_β_ phase at 15 °C was 6.13 nm. In addition to the L_β_ peaks from POPE/20 mol% C18-ceramide, a small reflection was observed at 4.42 nm (red arrow). This reflection can be attributed to the reflection from the solid C18-ceramide cluster excluded from the membrane. This reflection became evident at 55–65 °C and the first-, second- and third-order reflections were observed at 4.44 nm (S = 0.225 nm^−1^) (Cer1), 2.22 nm (S = 0.450 nm^−1^) (Cer2) and 1.48 nm (S = 0.676 nm^−1^) (Cer3), respectively. These reflections disappeared at 75 °C. This indicates the occurrence of ordering (recrystallization) and melting of C18-ceramide in the system. The L_α_ phase was observed between 25 °C and 55 °C, while the H_II_ phase appeared at 50 °C. Thus, the L_β_/L_α_ and L_α_/H_II_ phase transitions were partially merged at this C18-ceramide concentration. The *d*-spacing of the L_α_ phase at 45 °C was 5.21 nm.

At 30 mol% C18-ceramide, the *d*-spacings of the L_β_ phase at 15 °C and the L_α_ phase at 45 °C were 6.13 and 5.21 nm, respectively. These values were the same as those of POPC/20 mol% C18-ceramide. L_β_–L_α_ and L_α_–H_II_ phase transitions took place at the same temperatures as those of POPE/20 mol% C18-ceramide. Peak splitting, which suggests the presence of large-scale phase separation in the membrane, was not detected in either the L_β_ or L_α_ phase. The reflection from the C18-ceramide cluster became evident for POPE/30 mol% C18-ceramide and up to third-order refection was observed (reflections located at 4.42 nm (Cer1), 2.21 nm (Cer2) and 1.47 nm (Cer3)). The positions of these reflections were almost constant until 75 °C, indicating that the *d*-spacing of the C18-ceramide cluster was unaltered.

As mentioned above, only a single lamellar diffraction peak series appeared in the L_α_ phase at 45 °C even for POPE/C18-ceramide mixtures. The *d*-spacings slightly differed from those of pure POPE. However, reconstituted electron density profiles (Fig. S1) revealed nearly constant bilayer thicknesses.

### Wide-angle X-ray scattering (WAXS) measurements

**Figure 4:**
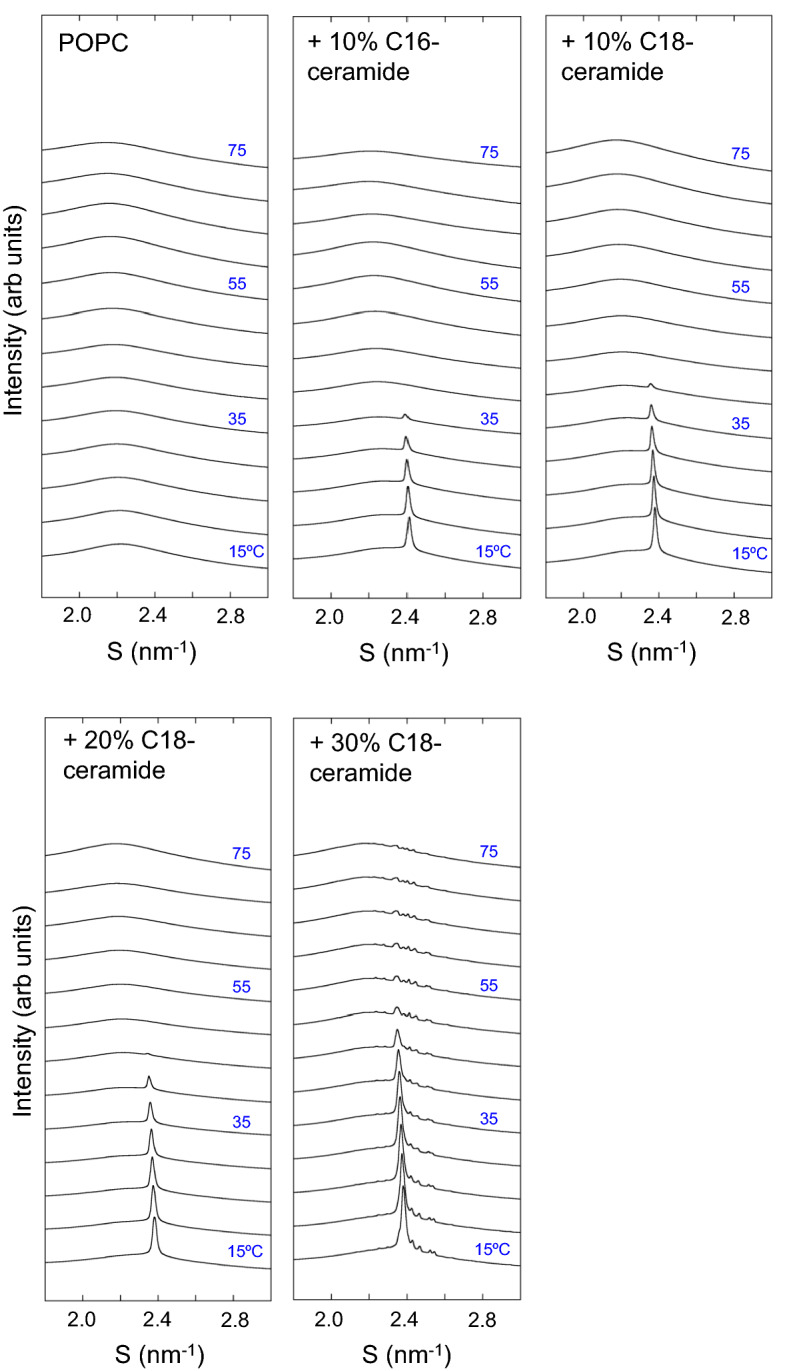
Wide-angle X-ray scattering (WAXS) profiles obtained from POPC, POPC/10% C16-ceramide and POPC/C18-ceramide containing different amounts of C18-ceramide (10, 20, 30 mol%). Data were taken from 15 to 75 °C at 5° interval. All samples (150 mM final lipid concentration) were dispersed in 20 mM HEPES buffer (pH 7.0) containing 100 mM NaCl and 10 mM EDTA.

Information on the acyl-chain packing can be obtained from WAXS. Figure 4 shows the WAXS profiles of the POPC dispersion. Over the temperature range of 15–75 °C, only a broad peak centered at ~ 0.45 nm, a characteristic profile for the melted acyl chains in L_α_ phase, was observed^62^. In the presence of 10 mol% C16-ceramide or C18-ceramide, a sharp symmetric peak at 0.420 nm (S = 2.38 nm^−1^), a characteristic of the L_β_ gel phase^62^, appeared over the temperature range of 15–35 °C (C16-ceramide) or 15–40 °C (C18-ceramide) in addition to the broad peak from the L_α_ phase (Fig. 4). This sharp reflection can be assigned to the membrane region rich in ceramide. The peak position of this reflection was slightly shifted to the small angle region with increasing temperature, indicating that the acyl-chain packing is loosened at higher temperatures. At 40 °C (C16-ceramide) or 45 °C (C18-ceramide) and above, and only the broad peak of the L_α_ phase was observed.

At 20 mol% C18-ceramide, the reflection from the ceramide-rich region was observed from 15–45 °C. The reflection position at 15 °C was 0.420 nm and disappeared at 50 °C (Fig. 4). In the temperature range of 15–45 °C, the broad peak of the L_α_ phase (corresponding to the C18-ceramide-poor region) decreased compared to POPC/10 mol% C18-ceramide (Fig. 4). This agrees well with the SAXS observation that the lamellar reflection intensity of the C18-ceramide-poor region was lower than that of the C18-ceramide-rich region (see Fig. 2).

In the case of POPC/30 mol% C18-ceramide (Fig. 4), in addition to the sharp reflection at 0.420 nm, several small reflections were observed at 0.444 nm (S = 2.25 nm^−1^), 0.435 nm (S = 2.30 nm^−1^), 0.412 nm (S = 2.43 nm^−1^), 0.405 nm (S = 2.47 nm^−1^), 0.397 nm (S = 2.519 nm^−1^) , and 0.393 nm (S = 2.545 nm^−1^) at 15 °C. Our samples were multilamellar vesicles that give diffraction peaks corresponding to the repeating lamellar structure observed in the small-angle region. In the L_β_ phase of lipids, diffraction peaks originating from a two-dimensional lattice of hydrocarbon chain packing are observed in the wide-angle region. In this case, only one or two diffraction peaks are usually observed in the wide-angle region of S = 2.0–3.0 nm^−1^. In contrast, crystals have a three-dimensional periodic structure. A larger number of periodic lengths determine the three-dimensional lattice of the crystals. Therefore, the appearance of multiple diffraction peaks is an evidence of the formation of crystals with a three-dimensional periodic structure. The presence of these peaks indicates the formation of an ordered crystal. To determine the space group of the crystal, it is necessary to detect diffraction peaks over a wider angle region. This is one of challenging future tasks.

Peaks of 0.45 nm, 0.41 nm, 0.40 nm and 0.38 nm are observed in the crystalline C16-ceramide at 68 °C^31^, strongly suggesting that the observed peaks are derived from C18-ceramide. Although the reflection intensity at 0.420 nm decreased with increasing temperature, the reflections corresponding to solid C18-ceramide (S = 2.25 nm^−1^, 2.30 nm^−1^, 2.43 nm^−1^, 2.47 nm^−1^, 2.519 nm^−1^ and 2.545 nm^−1^) were clearly observed up to ~ 65 °C, and decreased at 75 °C. In the corresponding DSC thermogram, an exothermic peak was observed at ~ 65 °C. Considering the WAXS data, the exothermic peak at approximately 65 °C could be interpreted as a transition of the phase-separated C18-ceramide from a metastable phase to a stable crystalline phase. Above 30 °C, the broad peak of the L_α_ phase became evident, as observed in POPC/20 mol% C18-ceramide.

**Figure 5:**
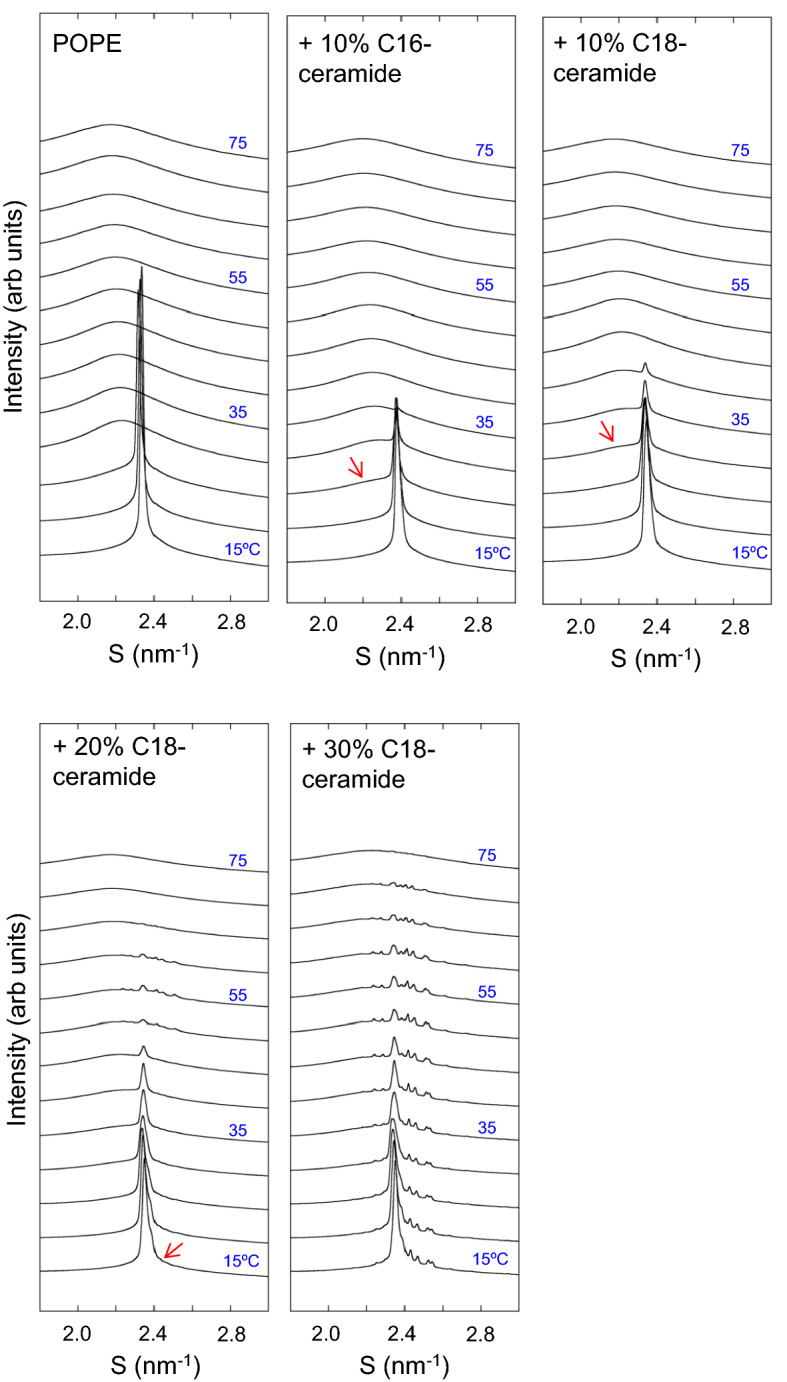
Wide-angle X-ray scattering (WAXS) profiles obtained from POPE, POPE/10% C16-ceramide and POPE/C18-ceramide containing different amounts of C18-ceramide (10, 20, 30 mol%). Data were taken from 15 to 75 °C at 5° interval. All samples (150 mM final lipid concentration) were dispersed in 20 mM HEPES buffer (pH 7.0) containing 100 mM NaCl and 10 mM EDTA.

The WAXS profile of POPE is shown in Fig. 5. A sharp reflection corresponding to the L_β_ phase appeared at 15–25 °C. At 15 °C, the acyl chain spacing of POPE was 0.428 nm (S = 2.336 nm^−1^), characteristic of the gel (L_β_) phase^63,64^. Above 30 °C, only a broad peak from disordered acyl chains (L_α_ phase) was observed. In contrast, the WAXS profile of POPE/10 mol% C16-ceramide showed an L_β_ phase up to 35 °C. In addition to the reflection from the L_β_ phase, a broad peak of the L_α_ phase appeared above 25 °C (red arrow). Two phases coexisted up to 35 °C. In POPE/10 mol% C18-ceramide, the L_β_ phase was observed up to 40 °C. At 30 °C, the L_α_ phase appeared (red arrow), and two phases coexisted up to 40 °C. The acyl chain spacings of POPE/10 mol% C16-ceramide and POPE/10 mol% C18-ceramide at 15 °C were 0.426 nm (S = 2.35 nm^−1^), which is smaller than that of POPE. At 40 °C, the acyl chain spacing was 0.428 nm (S = 2.34 nm^−1^).

For POPE/20 mol% C18-ceramide, reflection from the L_β_ phase was observed at 15 °C at 0.425 nm (S = 2.353 nm^−1^), with a shoulder peak (red arrow) on the wider angle side, suggesting the phase separation of the ceramide-rich and ceramide-poor regions. In addition, several small peaks were observed at almost the same positions as those observed in the POPC/20 mol% C18-ceramide system (0.448 nm (S = 2.233 nm^−1^), 0.440 nm (S = 2.275 nm^−1^), 0.412 nm (S = 2.427 nm^−1^), 0.405 nm (S = 2.469 nm^−1^), 0.397 nm (S = 2.519 nm^−1^) and 0.393 nm (S = 2.545 nm^−1^)) (Fig. S2). These peaks were evident in the temperature range of 50–60 °C, while above 65 °C, only a broad peak of the L_α_ phase was observed, indicating that recrystallization and melting of C18-ceramide occurred in this temperature range. At the 30 mol% C18-ceramide, reflections from solid C18-ceramide were clearly observed (Fig. S2). These reflections were evident up to 70 °C and largely disappeared at 75 °C, suggesting that the melting of solid C18-ceramide occurred at this temperature. It is conceivable that the observed endothermic transition at ~ 68- ~ 78 °C for POPE/30 mol% C18-ceramide in DSC corresponds to this WAXS observation. Although DSC thermograms clearly exhibited the exothermic transition at ~ 52–58 °C, that indicates the recrystallization of C18-ceramide for the samples containing 30 mol% C18-ceramide, the increase in the WAXS reflections originating from the C18-ceramide recrystallization was hardly detected (Fig. S2), and the reflections from solid C18-ceramide were still present. This discrepancy may be explained by the fact that the sample temperature was increased continuously in the DSC measurements, but the temperature was changed stepwise in the X-ray scattering measurements. Based on their DSC measurements, Shah et al.^31^ reported that the recrystallization behavior for C16-ceramide depends on the temperature scanning rates.

## Discussion

Ceramides exhibit a very high gel-to-liquid crystalline phase transition temperature^31,32^. In the DSC experiments, the presence of ceramide in PC results in the appearance of additional endotherms^35,47,55^. Similar to the 1,2-dipalmitoyl-*sn*-glycero-3-phosphocholine (DPPC)/C16-ceramide^35^, 1,2-dimyristoyl-*sn*-glycero-3-phosphocholine (DMPC)/C16-ceramide^55^ and POPC/brain ceramide^47^ systems, C18-ceramide induced broad endothermic peaks upon inclusion in POPC. These peaks shifted to higher temperatures with increasing concentrations of C18-ceramide. Our results are consistent with published results and indicate that ceramide-rich domains are formed in PC membrane^40,47,55^.

Similar to the DEPE/C16-ceramide^42,43^ and DOPE/C16-ceramide systems^44^, the addition of C18-ceramide to POPE broadened the L_β_/L_α_ phase transition, exhibiting a shoulder of the endotherm at higher temperatures. These results indicate the coexistence of mixtures of different lipid compositions in POPE/C18-ceramide. As observed previously in the C16-ceramide system^42–44^, C18-ceramide decreased the L_α_/H_II_ phase transition temperature of POPE. In contrast to POPC/C18-ceramide, there was no clear indication of the appearance of new endotherms in POPE/C18-ceramide.

The appearance of new peaks in SAXS and WAXS and the peak splitting in SAXS for the POPC/C18-ceramide membrane indicate the formation of ceramide-rich gel domains in POPC. The results of SAXS and WAXS are consistent with the DSC, which indicates the formation of a C18-ceramide-rich condensed gel domain in the POPC membrane. Above the phase transition temperature of the C18-ceramide-rich gel domain, the SAXS profiles of POPC/C18-ceramide still showed the coexistence of two lamellar phases with different *d*-spacings up to the highest temperature tested (75 °C) (Fig. 2). The difference in the *d*-spacings of these two phases increased with increasing temperature.

In contrast to POPC/C18-ceramide, the presence of 10 mol% C18-ceramide in POPE resulted neither in the appearance of a new peak nor the splitting of lamellar reflections in SAXS (Fig. 3), despite a reflection peak originating from the gel phase in the membrane observed with POPE/10 mol% C18-ceramide by WAXS (Fig. 5). It is possible that the *d*-spacings of the C18-ceramide-rich and -poor regions were equal and produced a single lamellar reflection, even if the two regions were completely phase-separated. However, even when C16-ceramide was employed instead of C18-ceramide, no splitting of lamellar reflection was observed by SAXS (Fig. 3), suggesting that long chain ceramides do not form distinct gel domains in POPE. This SAXS observation suggests that compared to POPC, more POPE molecules are solubilized in the C18-ceramide domain.

The observed difference in the effect of C18-ceramide on POPC and POPE may be due to the difference in the main phase transition temperature of POPC^47–50^ and POPE^44,51–54^ which is caused by the strong hydrogen bonding and small headgroup of PE.

Table 1 summarizes the DSC results of the phase transition temperatures of POPC and POPE at lower concentrations of C18-ceramide. Despite the ~ 30° difference in the main phase transition temperature of POPC and POPE, the L_β_–L_α_ phase transition temperature was very close between POPC/C18-ceramide and POPE/C18-ceramide. The endpoint temperature of the L_β_–L_α_ phase transition in POPC/C18-ceramide (95:5) was 41.4 °C, whereas that in POPE/C18-ceramide (95:5) was 37.6 °C. Table 2 indicates the lipid phases of POPC and POPE near physiological temperature in the presence of C16-ceramide and C18-ceramide. Table 2 summarizes the results of the present study and published results. Whereas the presence of 5 mol% C16-ceramide did not change the lipid phase of POPC and POPE between 35–40 °C, C18-ceramide induced phase separation of the membrane. However, a 1 mol% increase in C16-ceramide from 5 to 6% resulted in a phase-separated membrane^44^ (Table 2). Table 2 shows that small variations in the amount of long chain ceramides dramatically change the lipid phases of POPC and POPE at physiological temperature. Table 2 also indicates that C16-ceramide and C18-ceramide affect the lipid phases of POPC and POPE differently.

**Table 2:** Lipid phase of C16-ceramide or C18-ceramide-containing POPC and POPE.

	35 °C	36	37	38	39	40
POPC^a^	L_α_	L_α_	L_α_	L_α_	L_α_	L_α_
POPC + C16-ceramide 5%^b^	L_α_	L_α_	L_α_	L_α_	L_α_	L_α_
POPC + C18-ceramide 5%	L_α_ + L_β_	L_α_ + L_β_	L_α_ + L_β_	L_α_ + L_β_	L_α_ + L_β_	L_α_
POPC + C16-ceramide 10%^b^	L_α_ + L_β_	L_α_	L_α_	L_α_	L_α_	L_α_
POPC + C18-ceramide 10%	L_α_ + L_β_	L_α_ + L_β_	L_α_ + L_β_	L_α_ + L_β_	L_α_ + L_β_	L_α_ + L_β_
POPE^c^	L_α_	L_α_	L_α_	L_α_	L_α_	L_α_
POPE + C16-ceramide 5%^d^	L_α_	L_α_	L_α_	L_α_	L_α_	L_α_
POPE + C18-ceramide 5%	L_α_ + L_β_	L_α_ + L_β_	L_α_ + L_β_	L_α_	L_α_	L_α_
POPE + C16-ceramide 6%^d^	L_α_ + L_β_	L_α_ + L_β_	L_α_ + L_β_	L_α_	L_α_	L_α_
POPE + C16-ceramide 10%^d^	L_α_ + L_β_	L_α_ + L_β_	L_α_ + L_β_	L_α_ + L_β_	L_α_ + L_β_	L_α_ + L_β_
POPE + C18-ceramide 10%	L_α_ + L_β_	L_α_ + L_β_	L_α_ + L_β_	L_α_ + L_β_	L_α_ + L_β_	L_α_ + L_β_

References: ^a^^47–50^, ^b^^40^, ^c^^44,51–54^, ^d^^44^.

It has been reported that at the phase transition temperature, lipid reorganization such as flip-flop is accelerated^65^. Previously, Huang et al. showed that the addition of C16-ceramide or bovine brain ceramide to DPPC at 45 °C induces L_α_ and L_β_ phase separation and activates phospholipase A_2_ activity^34,66^. Our data and published results^36,37,40,41,44^ suggest that the activation of the enzyme may occur at physiological temperature in biomembranes in a ceramide chain length and content-dependent manner. It is proposed that membrane lipids contribute to the spatial organization of membrane proteins via the difference of lipid phase^67–69^. Previous results and our results suggest that ceramide-dependent signaling is triggered, at least in part, by altered partitioning of signal proteins in the membrane.

## Materials and methods

### Materials

1-Palmitoyl-2-oleoyl-*sn*-glycero-3-phosphocholine (POPC), 1-palmitoyl-2-oleoyl-*sn*-glycero-3-phosphoethanolamine (POPE), *N*-palmitoyl-d-erythro-sphingosine (C16-ceramide) and *N*-stearoyl-d-erythro-sphingosine (C18-ceramide) were purchased from Avanti Polar Lipids, Inc. (Alabaster, AL) and used without further purification.

### Differential scanning calorimetry (DSC)

Lipid samples were prepared for DSC as follows. A lipid film was formed from a chloroform solution of lipids evaporated under a stream of nitrogen gas, then dried in high vacuum overnight, hydrated and vortexed with buffer solution containing 20 mM 4-(2-hydroxyethyl)-1-piperazineethanesulfonic acid (HEPES), 100 mM NaCl, and 10 mM EDTA (pH 7.0). The final concentration of lipid was 1 mM. DSC measurements were performed with a Microcal VP-DSC microcalorimeter (MicroCal, Northampton, MA)^70^. Four successive DSC thermograms were recorded for each sample at a scan rate of 0.5°/min. For the L_β_–L_α_ phase transition of pure POPE bilayer, this rate has been reported to be sufficiently slow and that it can be regarded as quasi-static^56^. After the first one, heat scans on the same sample yielded superimposable thermograms. The 3rd scan of POPC, 4th scan of POPC/5% C18-ceramide, 4th scan of POPC/10% C18-ceramide, 3rd scan of POPC/20% C18-ceramide, 4th scan of POPC/30% C18-ceramide, 2nd scan of POPE, 3rd scan of POPE/5% C18-ceramide, 4th scan of POPE/10% C18-ceramide, 3rd scan of POPE/20% C18-ceramide and 3rd scan of POPE/30% C18-ceramide are shown. Data were plotted and analyzed with the Origin software package (OriginLab Corporation, Northampton, MA).

### Small- and wide-angle X-ray scattering

The dispersed lipid samples for X-ray measurements were prepared essentially as described for DSC measurements, with the exception that the concentration of lipid was 150 mM. To ensure homogeneous hydration of the samples, the hydrated samples were incubated at 75 °C for 5 min and cooled on ice for 5 min. This heating–cooling cycle was repeated at least three times before the X-ray measurements. Small-angle X-ray scattering (SAXS) and wide-angle X-ray scattering (WAXS) measurements were carried out at RIKEN Structural Biology Beamline I (BL45XU)^70–73^ at SPring-8, 8 GeV synchrotron radiation source, Hyogo, Japan. The X-ray wavelength used was 0.9 Å, and the beam size at the sample position was ~ 0.4 × 0.7 mm^2^. Samples were measured in a sample cell with a path length of 1.5 mm and a pair of thin quartz windows (20 μm thickness). The sample temperature was controlled to ± 0.01° with a high precision thermoelectric device. The SAXS was recorded with a 1–5 s exposure time by a beryllium-windowed X-ray image intensifier coupled with a cooled CCD camera (1000 × 1018 pixels). The required corrections were applied to the recorded images^74^. The WAXS patterns were recorded with a RIGAKU imaging plate detector (R-AXIS IV^++^, active area size: 30 by 30 cm^2^) with a 30 s exposure time. Buffer profiles were also measured at respective temperatures to subtract the background. The two-dimensional powder diffraction patterns were circularly averaged and reduced to one-dimensional profiles using FIT2D version 12.012 (http://www.esrf.fr/computing/scientific/FIT2D/), a 2-D data reduction and analysis program. The reciprocal spacing (*s*), *s* = 1/*d* = (2/*λ*)sin *θ* (where *d* is the lattice spacing, 2*θ* is the scattering angle, and *λ* is the wavelength of X-ray), was calibrated with silver behenate by the long-period spacing of 5.838 nm^75^.

### Electron density calculation

One-dimensional electron density profiles across the bilayer normal direction were calculated based on the lamellar diffraction intensities. After subtracting the background, the integrated intensities of each lamellar diffraction peak ($I(h)$) were estimated by fitting each $h$ order peak with a pseudo-Voigt function^76^. The structure amplitude ($F(h)$) was equal to $\sqrt{{h}^{2}I(h)}$, according to Blaurock and Worthington^77^. The relative electron density profile ($\rho \left(x\right)$) was calculated as follows;

1 $$\rho \left(x\right)=\sum_{h=1}^{h={h}_{\mathrm{max}}}\mathrm{exp}\phi (h)F\left(h\right)\mathrm{cos}\left(\frac{2\uppi hx}{d}\right)$$

where $d$ is the lamellar repeat spacing, $x$ is the distance from the center of the bilayer, and $\phi \left(h\right)$ is the phase angle for order $h$. The value of each $\phi \left(h\right)$ was chosen based on the results of previous studies on pure POPE bilayers^52,63^.

## Supplementary Information

Supplementary Figures.

## Data Availability

The datasets generated and/or analyzed during the current study are available from the corresponding author on reasonable request.

## Abbreviations

C16-ceramide: Palmitoyl ceramide
C18-ceramide: Stearoyl ceramide
DSC: Differential scanning calorimetry
H_II_: Inverse hexagonal
L_α_: Lamellar liquid crystalline
L_β_: Lamellar gel
POPC: 1-Palmitoyl-2-oleoyl-*sn*-glycero-3-phosphocholine
POPE: 1-Palmitoyl-2-oleoyl-*sn*-glycero-3-phosphoethanolamine
SAXS: Small-angle X-ray scattering
SM: Sphingomyelin
SMase: Sphingomyelinase
Tm: Phase transition temperature
WAXS: Wide-angle X-ray scattering
