# Supplementary material for: Long chain ceramides raise the main phase transition of monounsaturated phospholipids to physiological temperature
Source: Sci Rep. 2022 Dec 2;12:20803. doi: 10.1038/s41598-022-25330-y (PMC9718810; doi:10.1038/s41598-022-25330-y)
Supplement: Supplementary file 1 — Supplementary Figures. [file 41598_2022_25330_MOESM1_ESM.pdf]

Suppl Fig. 1
Takahashi et al.

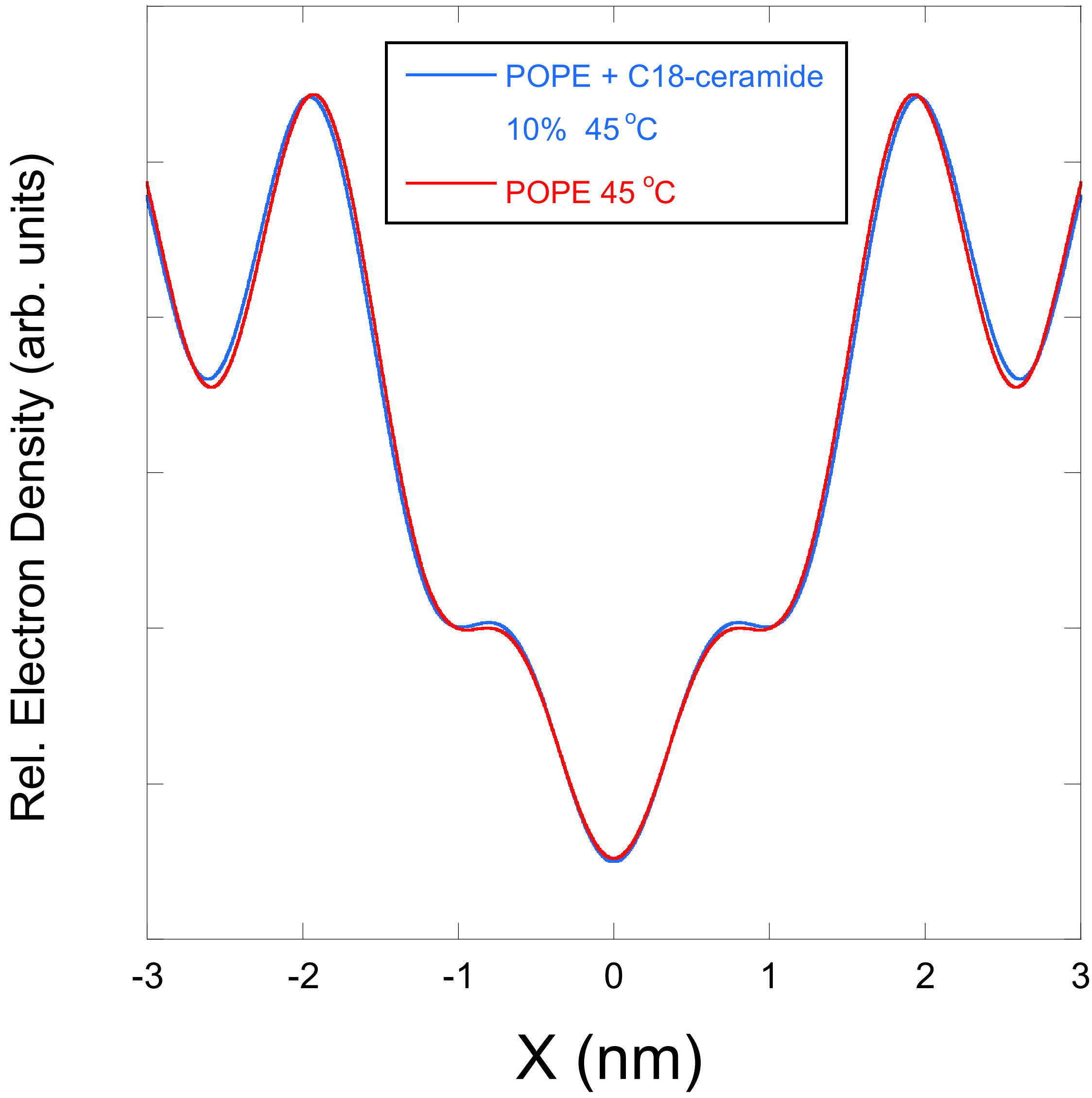

Fig.S1
Electron density profiles of pure POPE (red line) and POPE/C18-ceramide (blue line) systems at 45°C. Among the constituent atoms of phospholipids, the atom with the highest number of electrons is the phosphorus atom. Thus, the peak top positions correspond to that of phosphorus atom in the polar headgroup. The distance between the two highest peaks reflects the bilayer thickness. The addition of C18-ceramide did not significantly affect the thickness of the POPE bilayer membranes.

Suppl Fig. 2
Takahashi et al.

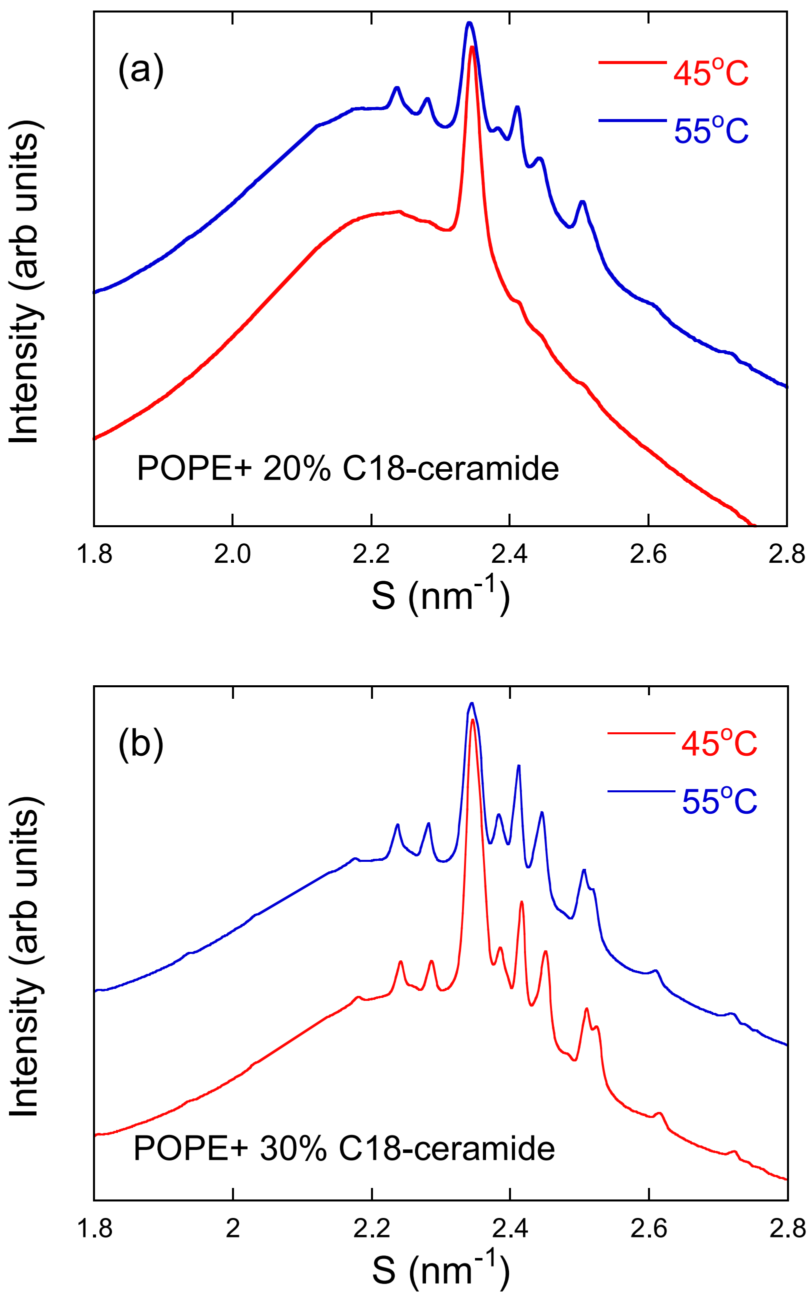

Fig.S2
Enlarged images of the WAXS profiles of POPE+20mol% C18-ceramide (a) and POPE+30mol% C18-ceramide (b) at 45°C (red lines) and 55°C (blue lines) shown in Fig. 5.
